# Microarray and qPCR Analyses of Wallerian Degeneration in Rat Sciatic Nerves

**DOI:** 10.3389/fncel.2017.00022

**Published:** 2017-02-10

**Authors:** Sheng Yi, Xin Tang, Jun Yu, Jie Liu, Fei Ding, Xiaosong Gu

**Affiliations:** Key Laboratory of Neuroregeneration of Jiangsu and Ministry of Education, Co-Innovation Center of Neuroregeneration, Nantong UniversityNantong, China

**Keywords:** Wallerian degeneration, peripheral nerve injury, microarray, gene expression, bioinformatic analysis

## Abstract

Wallerian degeneration occurs immediately following injury to mammal peripheral nerves. To better understand the molecular events occurring during Wallerian degeneration, a rat model of sciatic nerve transection was used to assess differentially expressed genes at 0.5, 1, 6, 12, 24 h, 4 days, 1, 2, 3, and 4 weeks post nerve injury (PNI). Hierarchical clustering, Euclidean distance matrix, and principal component analysis (PCA) collectively suggested three distinct phases within the post-injury period of 4 weeks. Gene ontology (GO) analysis suggested that phase I (0–6 h PNI), phase II (6–24 h PNI), and phase III (4 days to 4 weeks) were associated with acute response to injury, preformation of Wallerian degeneration, and complete execution of Wallerian degeneration, respectively. Critical signaling pathways and transcriptional factor networks responsible for the regulation of Wallerian degeneration were further identified and integrated using Kyoto Enrichment of Genes and Genomes (KEGG) pathway analysis and Ingenuity Pathway Analysis (IPA), respectively. Our results may help to elucidate some molecular mechanisms of gene regulation associated with Wallerian degeneration that occurs after traumatic injury to peripheral nerve axons in mammals.

## Introduction

Nerve injury is a common clinical occurrence with a steadily increasing incidence worldwide, and may cause life-long disability and impair quality of life (Gu et al., 2011; Lee et al., 2014). Following nerve injury, axons in the distal nerve stump are separated from neuronal cell bodies, and undergo Wallerian degeneration (Coleman, 2005). In the peripheral nervous system, Wallerian degeneration is typically detectable within 1–3 days after injury. Morphological analysis of the distal stumps of injured peripheral nerves showed that axon starts to disintegrate within hours after injury; proliferating Schwann cells and macrophages then engulf and clear the axon and myelin debris about three days later (Geuna et al., 2009).

However, in mammal central nerves, Wallerian degeneration initiates much slower (Vargas and Barres, 2007). Additionally, Wallerian degeneration rarely occurs in many invertebrates and lower vertebrates (Bittner et al., 2000, 2016). The different occurrences of Wallerian degeneration in different nervous systems make the role of Wallerian degeneration obscure, and thus many attempts have been made to investigate molecular basis underlying Wallerian degeneration.

A genetic study on the slow Wallerian degeneration (Wld^s^) mouse suggests that over-expression of nicotinamide mononucleotide adenylyltransferase fusion protein slows down Wallerian degeneration (Conforti et al., 2000; Mack et al., 2001). Another study reports that midkine knockout mice exhibit delayed axon degeneration and nerve regeneration following peripheral nerve injury (Sakakima et al., 2009). It has also been shown that activated potassium channel or inhibited sodium channel retards Wallerian degeneration of injured Drosophila axons (Mishra et al., 2013). And sterile alpha and TIR motif containing 1 (SARM1), a Toll-like receptor, has been demonstrated as an essential factor for triggering axon degeneration (Gerdts et al., 2013; Loreto et al., 2015). Despite these interesting findings, much is still unknown about the molecular mechanisms involved in Wallerian degeneration. A systems-level analysis may help to obtain a global understanding of Wallerian degeneration from the perspective of gene regulation.

Microarray analysis has been widely adopted to characterize many differentially expressed genes in normal versus experimental conditions. In our previous studies, we performed microarray to identify differentially expressed genes in the distal nerve stump following sciatic nerve injury (Yao et al., 2012, 2013). By using the R software platform and the limma package, we were able to re-annotate and re-analyze these previous obtained microarray data. In an earlier study, we used Ingenuity Pathway Analysis (IPA) software program to analysis dynamic molecular changes in the injured distal nerve stump and gained some insights into Wallerian degeneration (Yu et al., 2016). In the current study, we made the joint use of Euclidean distance calculation, hierarchical clustering, principal component analysis (PCA), Gene ontology (GO) analysis, Kyoto Enrichment of Genes and Genomes (KEGG) pathway analysis, and IPA to further access genetic changes occurring during Wallerian degeneration.

## Materials and methods

### Animal surgery

Adult male Sprague-Dawley (SD) rats were obtained from the Experimental Animal Center of Nantong University. All animal procedures were performed in accordance with Institutional Animal Care guideline of Nantong University and ethically approved by the Administration Committee of Experimental Animals, Jiangsu Province, China. Animals were anesthetized by injection of mixed narcotics (85 mg/kg trichloroacetaldehyde monohydrate, 42 mg/kg magnesium sulfate, and 17 mg/kg sodium pentobarbital), and underwent surgical transection of sciatic nerves as previously described (Yu et al., 2012). Briefly, the rat sciatic nerve was lifted through an incision on the lateral aspect of the mid-thigh of the left hind limb, and a 10-mm nerve segment was excised. Rats were then randomly divided into ten groups according to different time points post nerve injury (PNI). Rats were sacrificed by decapitation at 0.5, 1, 6, 12, and 24 h, 4 days, and 1, 2, 3, and 4 weeks PNI and the distal nerve stumps were collected, respectively. Rats in control group were sham-operated and immediately (designated as 0 h PNI) sacrificed by decapitation.

### Microarray analysis

Total RNA was extracted using Trizol (Life technologies, Carlsbed, CA) according to the manufacturer's instructions. Contaminating DNA was removed using RNeasy spin columns (Qiagen, Valencia, CA). The quality of isolated RNA samples was evaluated with an Agilent Bioanalyzer 2100 (Agilent technologies, Santa Clara, CA) and the purified RNA was quantified using a NanoDrop ND-1000 spectrophotometer (Infinigen Biotechnology Inc., City of Industry, CA). An Affymetrix GeneChip Hybridization Oven 640 and Gene Array Scanner 3000 were used to perform microarray analysis. The R software (v.2.13.0) platform was applied to analyze the microarray data, and the limma (linear regression model) package was used to statistically analyze differentially expressed genes (Ritchie et al., 2015; Xu and Sun, 2015). The expression levels of mRNAs at each time point were compared with control. Genes having a fold change > 2 or < −2 and an adjusted *p* < 0.05 were considered as differentially expressed.

### Bioinformatic analysis

Bioinformatic analysis tools, including Euclidean distance calculation, hierarchical clustering, PCA, GO, KEGG, and IPA, were applied to investigate differentially expressed genes PNI. Briefly, Euclidean distance calculation was performed using the HeatMapImage GenePattern module, and hierarchical clustering was computed with the Euclidean distance measure using the Hierarchical Clustering module from GenePattern. PCA was performed using “Population PCA” tool from Harvard Medical School. Database for Annotation, Visualization, and Integrated Discovery (DAVID) bioinformatic resources were used to systematically screen differentially expressed genes and to enrich significant GO categories and KEGG pathways (Huang da et al., 2009a,b). IPA was performed to identify and connect differentially expressed transcription factors.

### Quantitative real time PCR (qPCR)

A total amount of 0.5 μg RNA samples were used as templates and reverse transcribed to cDNA using the Prime-Script reagent Kit (TaKaRa, Dalian, China). PCR was performed using SYBR Green Premix Ex Taq (TaKaRa) with specific primer pairs on an Applied Biosystems Stepone real-time PCR System. The sequences of primer pairs were as follows: FOSL1 (forward) 5′-GACCTCTGACCTATCCCCAGT-3′ and (reverse) 5′-GTCGTAGCTCTGCTCTGTGT-3′; SS18L1 (forward) 5′-TGCAGAGCCCATGAGTCAAC-3′ and (reverse) 5′-TCATCCTCCAACTTGTCGGTC-3′; and GAPDH (forward) 5′-CCTTCATTGACCTCAACTACATG-3′ and (reverse) 5′-CTTCTCCATGGTGGTGAAGAC-3′. The thermal cycler program was as follows: 5 min at 95°C; 40 cycles of 30 s at 95°C, 45 s at anneal temperature, and 30 s at 72°C; and 5 min at 72°C. Relative quantification of mRNA was conducted using the comparative 2^−ΔΔCt^ method with GAPDH as the reference gene.

### Statistical analysis

All data are expressed as means ± SEM, and analyzed by using GraphPad Prism 6.0 (GraphPad Software, Inc.). Differences between groups were tested using Student's *t*-test or one-way ANOVA.

## Results

### Differentially expressed genes in the distal nerve stump following sciatic nerve transection

**Figure 1:**
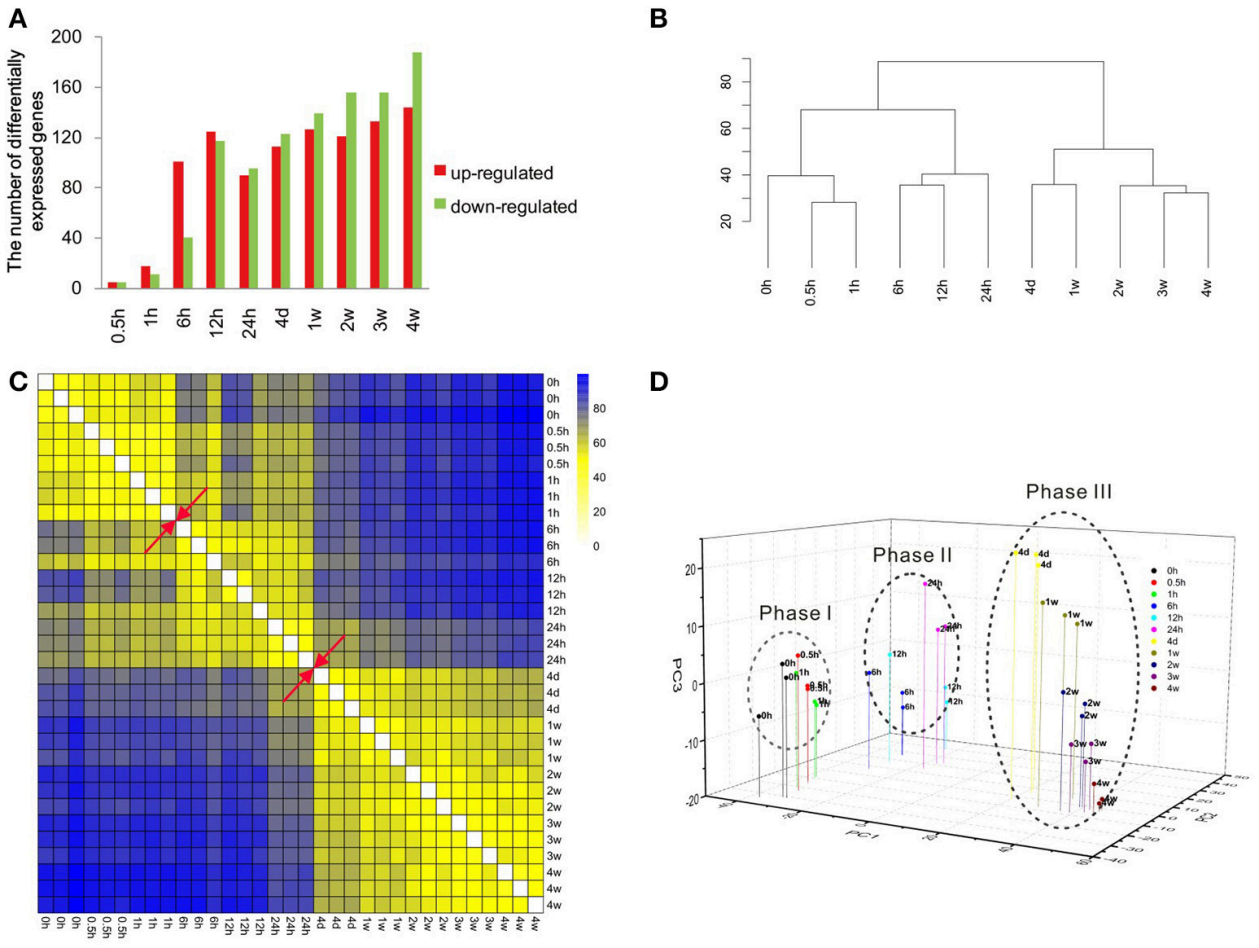
**Overview of the transcriptional pattern at the distal stumps following sciatic nerve transection. (A)** The bar graph showing the number of up-regulated (red) genes and down-regulated (green) genes at each time point following nerve transection as compared to control. **(B)** Euclidean distance heatmap, **(C)** hierarchical clustering, and **(D)** principal component analysis of differentially expressed genes at different time points following nerve transection.

Previous obtained microarray data (Yao et al., 2012, 2013) were re-annotated and re-analyzed to identify the expression patterns of more than 30,000 genes. The expression levels of these genes at different time points PNI were compared with control (the expression level at 0 h PNI) and differentially expressed genes were determined according to fold change (> 2 or < −2) and adjusted *p* < 0.05 (Table S1). Only a few genes were differentially expressed at 0.5 and 1 h PNI. More genes were differentially expressed at the ensuing time points PNI with a maximum number of differentially expressed genes being about 300 at 4 weeks PNI (Figure 1A). The most up-regulated gene was elevated for more than 13-folds (log_2_ ratio > 3.7) while the most down-regulated gene was reduced to ~1/30 (log_2_ ratio < −4.9). Top 10 up-regulated or down-regulated genes at each time point were listed in Table 1.

**Table 1:** **Top differentially expressed genes**.

**Gene**	**Time after injury**									
	**0.5 h**	**1 h**	**6 h**	**12 h**	**24 h**	**4 days**	**1 week**	**2 weeks**	**3 weeks**	**4 weeks**
Up-regulated	IL6	IL6	ERAL1	ERAL1	VGF	PCSK1	PCSK1	PCSK1	PCSK1	PCSK1
	CXCL2	PLAUR	PLAUR	MMP9	UCN2	TAF1D	SLC14A1	SLC14A1	KCNE1	KCNE1
	GULO	CCL20	KCNH7	IL1RN	MMP9	SLC14A1	MOXD1	MOXD1	SLC14A1	MOXD1
		CSF2	TAF1D	TAF1D	IL1RN	CACHD1	ST6GALNAC2	BDNF	MOXD1	SLC14A1
		CXCL3	RGD1309995	VGF	PTGER2	MMP9	MMP9	TAF1D	BDNF	BDNF
		IGSF21	IL1RN	RGD1309995	SLC14A1	ST6GALNAC2	TAF1D	REM2	MMP9	ARPP21
		EPHA4	SLC14A1	CXCL3	GMEB2	UCN2	BDNF	CACHD1	REM2	REM2
		CXCL2	MMP9	PTGER2	PCSK1	REM2	REM2	MMP9	CRYBB1	PPP1R17
		GRB7	IL6	UCN2	CLCA4L	XCL1	UCN2	ST6GALNAC2	GRIK1	GRIK1
		SLC16A13	PTGER2	PLAUR	RGD1305627	TTK	VGF	TCF23	BLDN6	TAF1D
Down-regulated	SLC9A1	UBTF	RFX4	PRICKLE2	SNRNP40	FOXA3	SLC7A14	CDH8	ZFP532	PVALB
	STEAP2	UBA7	ESRRG	LOC102546858	PCDH10	PDE6A	CHRNA3	CDK5R2	RGD1308742	GADL1
	RGD1305014	LOC100911508	RGD1307235	MSX2	ESRRG	CCDC37	LOC102553190	RERE	AQP3	KCNA1
	BTC	RGD130514	PCDHB17	KNDC1	SFTPA1	LOC10090909409	LOC102553043	GNMT	KCNA1	PEX5L
		RECQL4	PRL8A2	DMRT2	SHROOM1	MYH3	TRIM9	PRRT4	PEX5L	LOC102553868
			COL25A1	FSTL4	RGD1309108	PEX5L	C1QTNF4	PEX5L	F9	NRSN1
			TAS2R121	RAPGEFL1	GRIA2	AAED1	LECT1	C1QTNF4	DRD5	PEX5L
			SS18L1	PRRT2	DNAH7	PEX5L	UBQLN2	DCDC2	VOM1R100	GPR176
			CLIC3	PRR15L	P2RX5	ST8SIA2	LOC679158	SEL1L3	RLBP1	VOM2R75
			HPGD	ACVR1C	APBA2	RYR3	MCPT3	GALNT13	PEX5L	LMOD3

*The top 10 up-regulated and down-regulated genes based on fold change are listed*.

### Three transcriptional phases of Wallerian degeneration

Cluster analysis was performed to compare the similarity in gene expression profiles among different time points PNI. Data from hierarchical clustering suggested that the gene expression profiles could be separated into two major groups: one at 0–24 h PNI and another at 24 h to 4 weeks PNI (Figure 1B). In the first major group, 0, 0.5, and 1 h PNI were similar and 6, 12, and 24 h PNI were similar. In the second major group, 4 days and 1 week PNI were similar and 2, 3, and 4 weeks were similar (Figure 1B). Euclidean distance matrix showed three different phases following nerve injury; namely 0, 0.5, and 1 h PNI for phase I; 6, 12, and 24 h PNI for phase II; 4 days, 1, 2, 3, and 4 weeks for phase III (Figure 1C). PCA analysis suggested that the 4 weeks PNI could be clustered into three different phases (Figure 1D). More detailed analysis indicated that within phase III, 4 days and 1 week PNI seemed to be closer, while 2, 3, and 4 weeks PNI seemed to be closer. This result was consistent with that from hierarchical clustering (Figures 1B,D). Since intra-phase differences were not as obvious as inter-phase differences, the time period of 0 h to 4 weeks PNI was arbitrarily divided into three phases for the sake of studying Wallerian degeneration.

### Critical cellular components, molecular functions, and biological processes involved in Wallerian degeneration

DAVID database was used to annotate GO terms related to differentially expressed genes. The GO analysis helped to identify highly enriched categories of cellular components, molecular functions, and biological processes.

**Figure 2:**
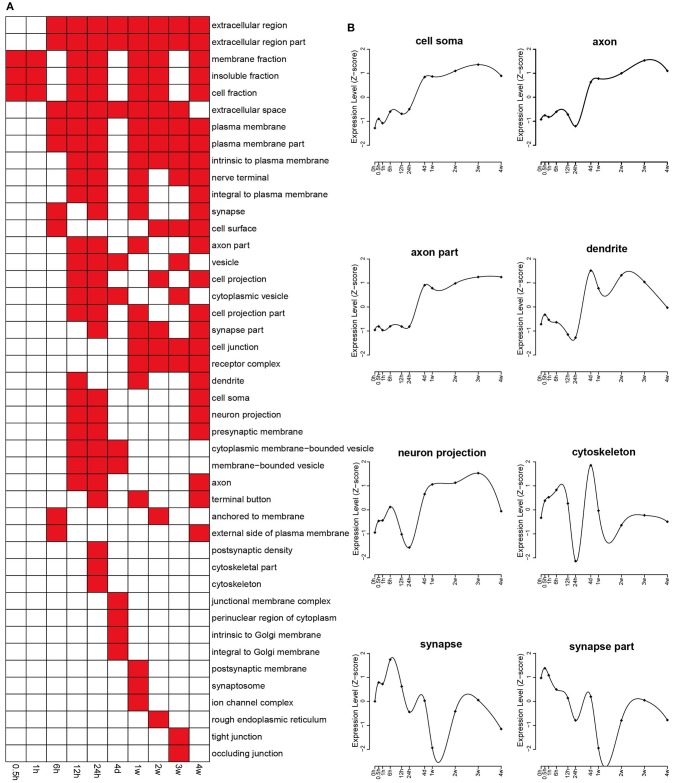
**Enriched GO cellular component of differentially expressed genes. (A)** GO cellular components with a *p* < 0.05 were labeled in red while cellular components with a *p* > 0.05 were labeled in white. **(B)** The expression profiles of differentially expressed genes involved in major cellular components related with the nervous system.

The enriched categories of cellular components at different time points PNI were identified with the *p*-value threshold set at 0.05 (Figure 2A). In brief, the categories of membrane fraction, insoluble fraction, and cell fraction were enriched at 1 h PNI, and the categories of extracellular region (part), extracellular space, and plasma membrane (part) were enriched starting from 6 h to 4 weeks PNI. To better analyze critical cellular components involved in nerve regeneration, the expression levels related to categories of cell soma, axon (part), dendrite, neuron projection, cytoskeleton, and synapse (part) were examined. The expression levels related to cell soma, axon (part), dendrite, and neuron projection were exponentially increased between 24 h and 4 days PNI and kept at a relatively high level at the ensuing time points PNI, while the expression changes related to synapse (part) seemed to lag several time points behind those related to other cellular components, showing a curved shape with alternate peaks and troughs (Figure 2B).

**Figure 3:**
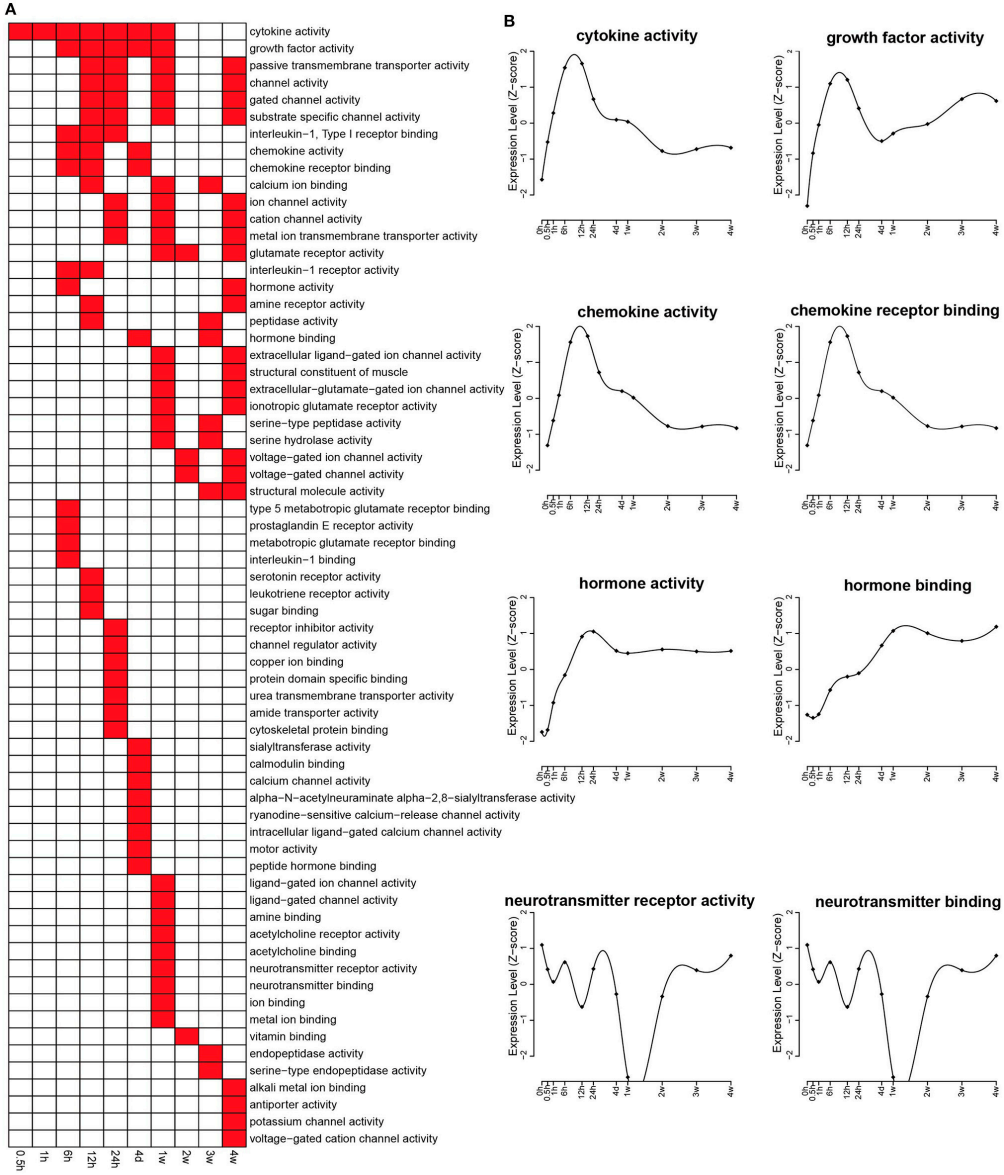
**Enriched GO molecular functions of differentially expressed genes. (A)** GO molecular functions with a *p* < 0.05 were labeled in red while molecular functions with a *p* > 0.05 were labeled in white. **(B)** The expression profiles of differentially expressed genes involved in several critical molecular functions related with nerve repair and regeneration.

Molecular functions related to differentially expressed genes were also identified by GO analysis according to *p* < 0.05, and the results were shown in Figure 3A. The expression changes related to some categories that are important for nerve regeneration, including cytokine activity, growth factor activity, chemokines activity, chemokines receptor binding, hormone activity, and hormone binding, were analyzed. These key categories were significantly enriched following nerve injury, reaching a peak at about 12 h PNI (Figure 3B). Similar to the expression changes related to cellular component categories of synapse (part), the expression changes of differentially expressed genes involved in two categories of neurotransmitter receptor activity and neurotransmitter binding also showed a curved shape (Figure 3B).

**Figure 4:**
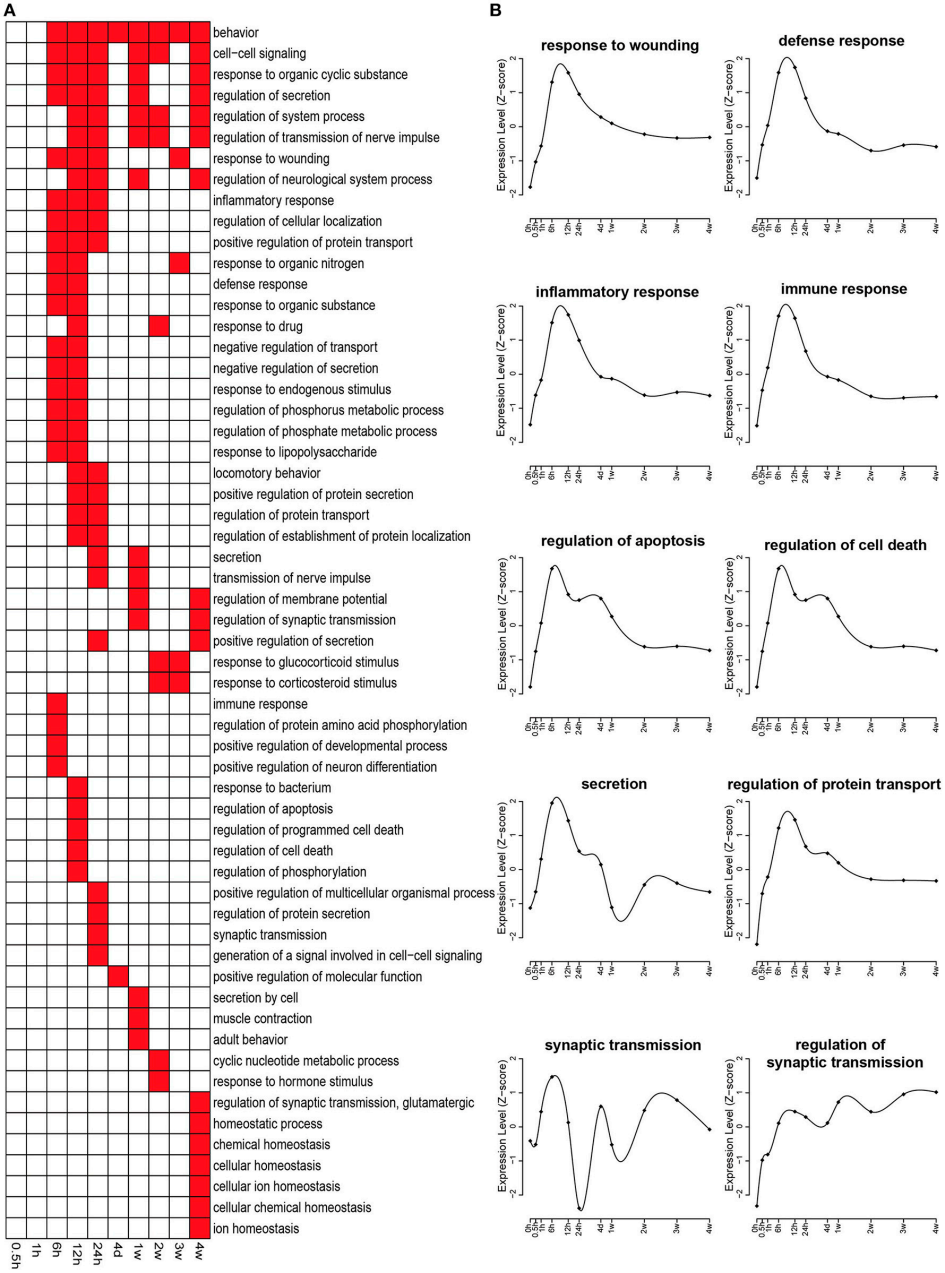
**Enriched GO biological processes of differentially expressed genes. (A)** GO biological processes with a *p* < 0.001 were labeled in red while biological processes with a *p* > 0.001 were labeled in white. **(B)** The expression profiles of differentially expressed genes involved in several critical biological processes during nerve repair and regeneration.

Biological processes related to differentially expressed genes were also annotated by GO analysis. A relatively larger number of significant biological processes with a *p* < 0.05 were involved, and therefore only top enriched biological processes with a *p* < 0.001 were displayed in Figure 4A. In an early stage (0.5 and 1 h PNI), few biological process categories were enriched. Starting from 6 h PNI, many biological process categories, such as response to wounding, defense response, inflammatory response, immune response, regulation of apoptosis, and regulation of cell death, were enriched (Figures 4A,B). These enriched categories might contribute to the onset of Wallerian degeneration. In the later stage, especially at 4 weeks PNI, several biological process categories, such as homeostatic process, chemical homeostasis, cellular homeostasis, cellular ion homeostasis, and ion homeostasis, were significantly enriched, suggesting that Wallerian degeneration was close to the end and a homeostatic state was reached (Figure 4A).

### Critical signaling pathways involved in Wallerian degeneration

**Figure 5:**
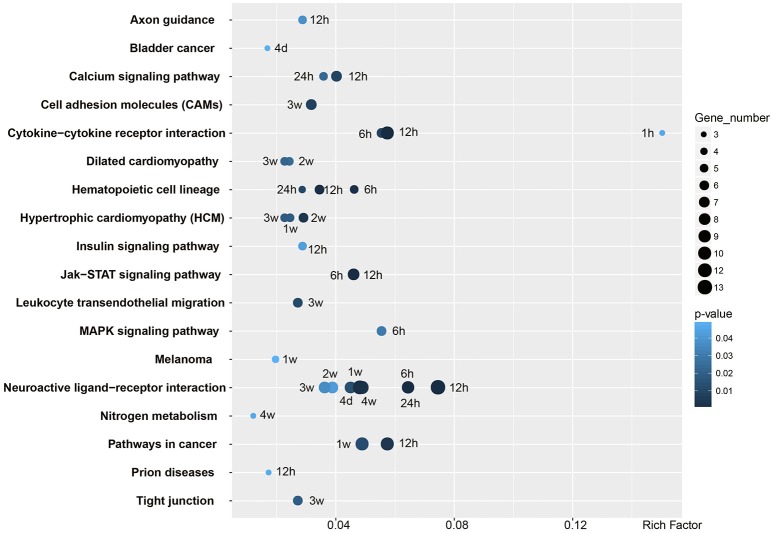
**Enriched KEGG pathways of differentially expressed genes**. KEGG pathways with a *p* < 0.05 are listed. The X-axis showed Rich Factor, the ratio of differentially expressed gene numbers to all gene numbers annotated in the KEGG pathway. The number of differentially expressed gene annotated in the specific KEGG pathway was expressed as the size of the circle. The *p*-value of KEGG pathway was corrected to *Q*-value ranging from 0 to 1.

GO analysis provided an overall insight into the cellular and molecular regulation of Wallerian degeneration. KEGG analysis was further performed to identify critical signaling pathways in the distal nerve stump at each time point (Figure 5). Two signaling pathways, cytokine-cytokine receptor interaction and neuroactive ligand-receptor interaction, were significantly involved in phases I and II of Wallerian degeneration, respectively. Cytokine-cytokine receptor interaction was activated as early as 1 h PNI and kept activated until 12 h PNI, while neuroactive ligand-receptor interaction was activated from 6 h to 4 weeks PNI. Besides these two pathways, calcium signaling pathway, insulin signaling pathway, Jak-STAT signaling pathway, and MAPK signaling pathway were also significantly involved in the process of Wallerian degeneration.

### Regulatory networks of differentially expressed transcription factors during Wallerian degeneration

**Figure 6:**
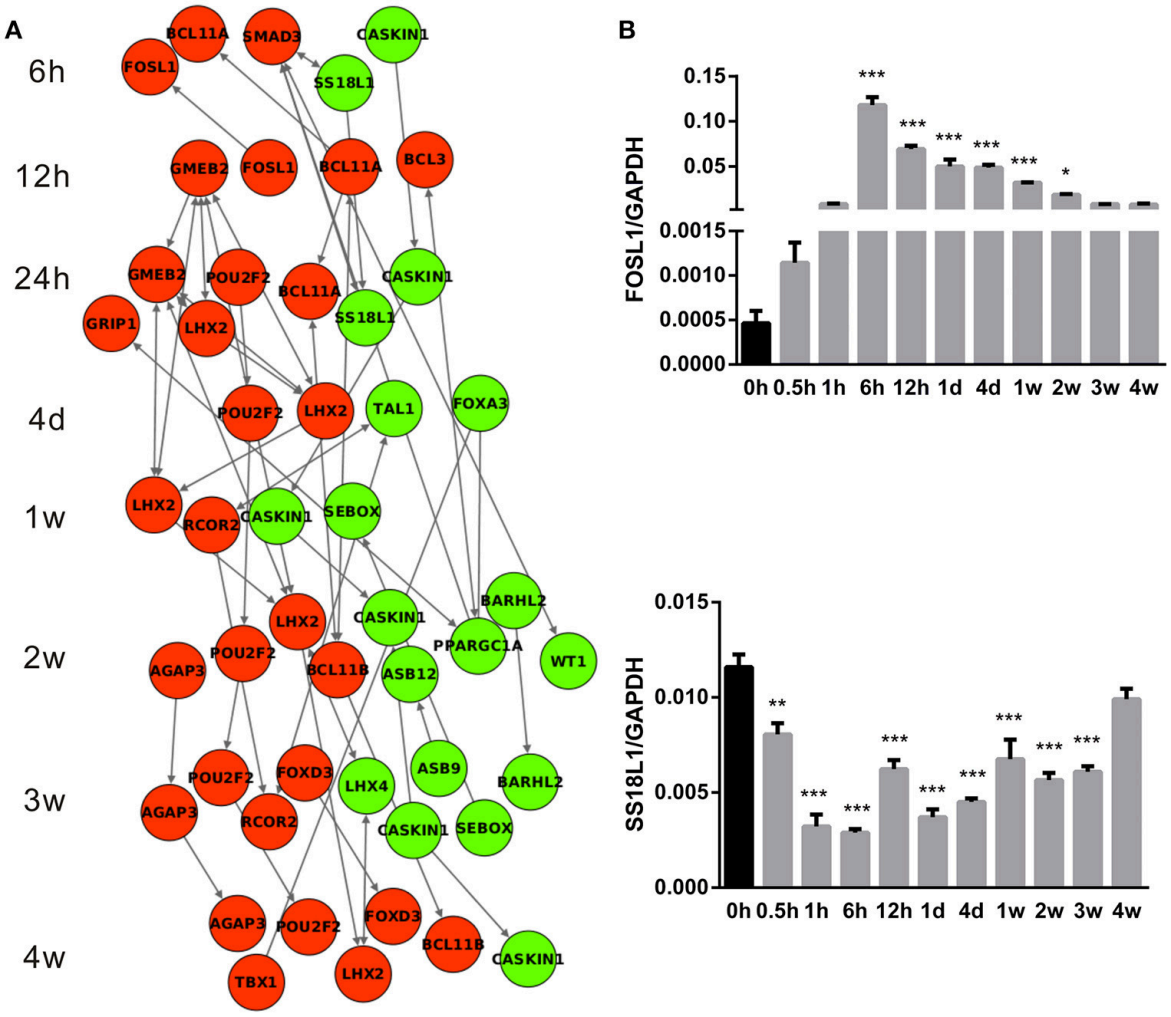
**Cascade network of differentially expressed transcription factors. (A)** The interaction network of up-regulated (red) transcription factors and down-regulated (green) transcription factors at each time point following nerve transection. **(B)** qPCR determination for mRNA expressions of FOSL1 and SS18L1. The relative level was normalized to GAPDH. The data, obtained from 3 independent experiments, are expressed as means ± SEM. ^*^p < 0.05, ^**^p < 0.01, and ^***^p < 0.001.

Differentially expressed transcription factors were specifically screened out as they might play central roles in the regulation of Wallerian degeneration and robustly affect diverse biological processes. IPA database was further used to construct the regulatory networks of differentially expressed transcription factors (Figure 6A). Consistent with previous results that few changes were observed in an early stage of Wallerian degeneration (Figure 1A), there were no differentially expressed transcription factors at 0.5 and 1 h PNI. Starting from phase II, the expression levels of several transcription factors were significantly changed. At 6 h PNI, FOSL1, BCL11A, SMAD3 were up-regulated, while SS18L1 and CASKIN1 were down-regulated. Moreover, these three transcription factors initiated the cascade sequence of transcriptional factors to accompany and modulate Wallerian degeneration. The temporal expression profiles of FOSL1 and SS18L1 were further validated by qPCR. Results from qPCR analysis were in agreement with the data from microarray analysis (Figure 6B).

## Discussion

Wallerian degeneration consists of a series of complicated cellular and molecular events and plays important roles in responses to nerve injury and perhaps to regeneration. Since its description by Waller in 1850 (Waller, 1850), many morphological studies have described events in Wallerian degeneration. In the current study, we systematically examined differentially expressed genes in the distal sciatic nerve stump at different time points following transection, aiming to have a better understanding of Wallerian degeneration from the genetic aspect.

Hierarchical clustering, Euclidean distance calculation, and PCA analysis results showed that the period of 0 h to 4 weeks PNI could be divided to three distinct phases by similarity of gene expression profiles among different time points. GO annotation was used to correlate the differentially expressed genes with cellular components, molecular functions, and biological processes. Enriched categories of cellular components, molecular functions, and biological processes at different time points were further studied in terms of three transcriptional phases.

**Figure 7:**
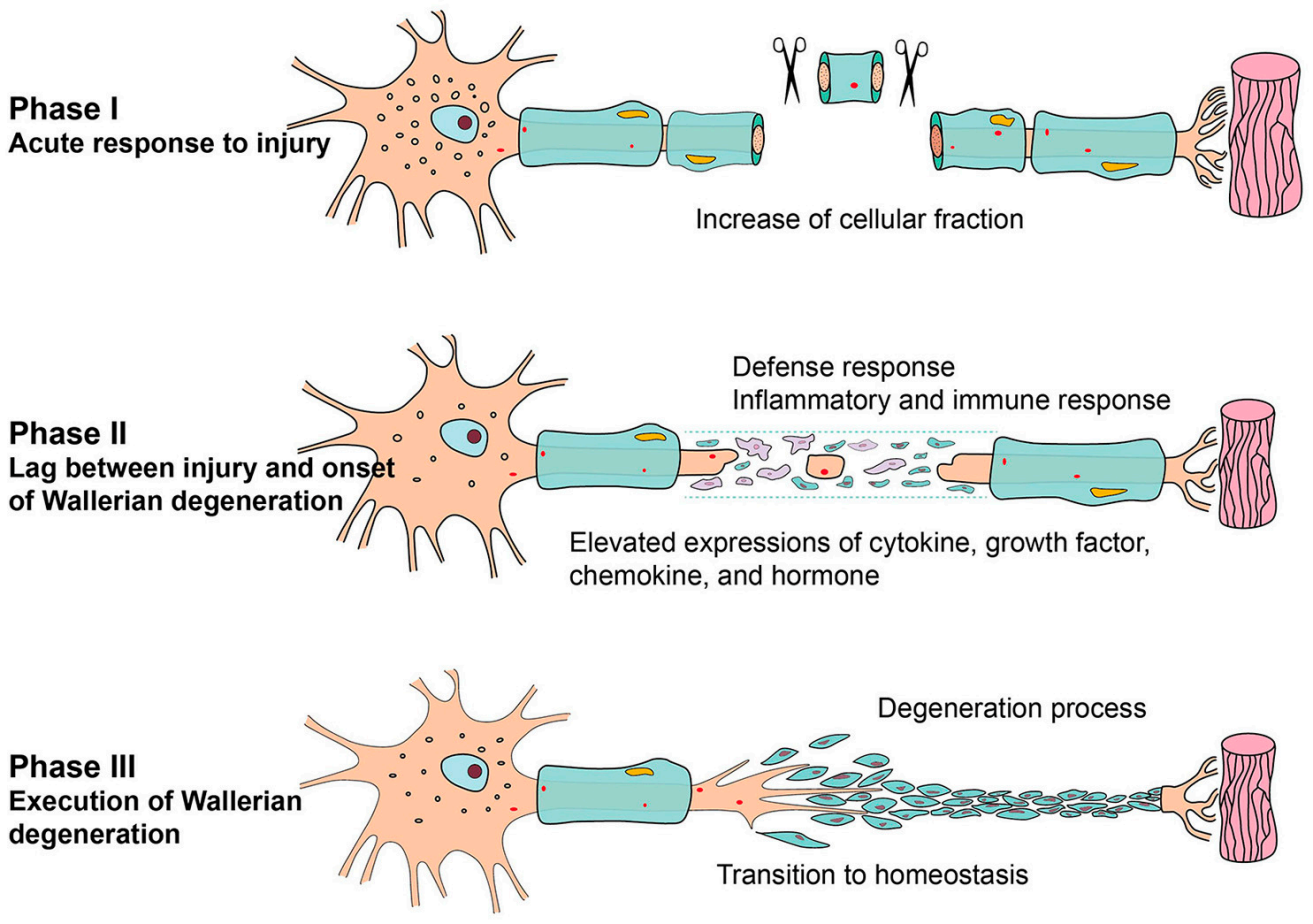
**Schematic diagram of the transcriptional changes during Wallerian degeneration**.

In phase I, only a few genes were differentially expressed, and not many GO categories were altered correspondingly. GO analysis also indicated that the most remarkable change in this phase might be cellular fraction enrichment. Accordingly, observations from photomicrograph suggested that neurofilament and myelin disintegrate into fragments at 1 h post injury (Sta et al., 2014). In phase II, relatively more numerous genes were differentially expressed, and more numerous GO categories were involved. Stimulus response-associated biological processes, such as response to wounding, defense response, inflammatory response, immune response, regulation of apoptosis, and regulation of cell death, were significantly activated. Increased inflammatory and immune response identified by bioinformatic analysis were consistent with morphological observations that phagocyte accumulated and infiltrated in the injured nerves (Sta et al., 2014). Activity/binding of cytokine, growth factor, chemokines, and hormone were significantly activated in this phase. In phase III, many biological processes were first activated, and then gradually declined to a homeostatic state. Taken together, the results showed that the time period PNI might be divided into three different phases, which corresponded to the formation and characteristics of Wallerian degeneration. In short, acute response to nerve injury, pre-formation of Wallerian degeneration, and comprehensive execution of Wallerian degeneration occurred in phase I (0–1 h PNI), phase II (6–24 h PNI), and phase III (within 4 days to 4 weeks PNI), respectively (Figure 7).

Enriched signaling pathways during Wallerian degeneration were also examined by KEGG analysis. Consistent with our previous studies (Li et al., 2013, 2014; Yao et al., 2013), cytokine-cytokine receptor interaction was activated in an early stage of Wallerian degeneration. Neuroactive-ligand receptor interaction was also significantly activated. Elevated activity of neuroactive-ligand receptor interaction signaling pathway has been linked with Parkinson's disease, a neurodegenerative disease (Hamza et al., 2011; Kong et al., 2015). Our current finding that neuroactive-ligand receptor interaction signaling pathway was activated during Wallerian degeneration might provide some preliminary evidence for the similarity between Wallerian degeneration and neurodegenerative disease. Besides these two most enriched signaling pathways, some other enriched pathways were also identified; for example, calcium signaling pathway. Calcium has long been implicated in Wallerian degeneration (Schlaepfer, 1974). Wld^s^ mouse exhibits an increased mitochondrial flux and an enhanced mitochondrial calcium buffering compared with normal mouse, suggesting the important roles of calcium in Wallerian degeneration (Coleman, 2005; Avery et al., 2012; Freeman, 2014). The current study, from the genetic level, demonstrated the involvement of calcium signaling in Wallerian degeneration, especially at 12 and 24 h PNI.

Transcription factors are DNA-binding proteins able to control the transcription rate of target genes (Karin, 1990; Latchman, 1997). Differentially expressed transcription factors mediate various physiological and pathological conditions through affecting the expression levels of target genes. They are usually incorporated in activation of multiple intracellular signaling cascades, interlinking to create the complex cascade networks (Patodia and Raivich, 2012). Based on their significance, differentially expressed transcription factors and their cascade networks were investigated in the current study. Further studies will be performed to determine the biological effects of these transcription factors on Wallerian degeneration.

## Conclusions

In the current study, we generated an integrated global view of gene expression patterns at the distal nerve stump following peripheral nerve injury. Bioinformatic analysis further enabled us to divide the phases and gain a comprehensive view of molecular changes during Wallerian degeneration.

## Author contributions

Conceptualization: SY, XT, FD, XG; methodology: SY, XT, JY; software: SY, XT, JY; validation: SY, XT, JY; formal analysis: SY, XT, JY; investigation: SY, XT, FD XG; resources: SY, XT, FD, XG; data curation: SY, XT, XG; writing (original draft preparation): SY, XT, JL; writing (review and editing): SY, XT, JL; visualization: SY, XT; supervision: FD, XG; project administration: FD, XG; and funding acquisition: SY, XT, FD, XG.

## Funding

This study was supported by National Natural Science Foundation of China (81370043), the Natural Science Foundation of Jiangsu Province, China (Grant No. BK20150409); Natural Science Foundation of Jiangsu Higher Education Institutions of China (Grant No. 15KJB180013); Natural Science Foundation of Nantong (Grant No. MS12015043); and a Project Funded by the Priority Academic Program Development of Jiangsu Higher Education Institutions (PAPD).

### Conflict of interest statement

The authors declare that the research was conducted in the absence of any commercial or financial relationships that could be construed as a potential conflict of interest.

## Supplementary material

Table S1 **Gene expression profiles at the distal stumps at each time point following sciatic nerve transection**. Gene expression values at each time point were listed and their expressions at 0.5, 1, 6, 12, 24 h, 4 days, 1, 2, 3, and 4 weeks post nerve injury were compared with 0 h control. Genes with the absolute value of fold change > 2 (the absolute value of log_2_FC > 1) and an adjusted p-value < 0.05 were considered as differentially expressed.
